# Long-Term Prognostic Significance of Three-Dimensional Speckle-Tracking Echocardiography-Derived Left Ventricular Twist in Healthy Adults—Results from the MAGYAR-Healthy Study

**DOI:** 10.31083/j.rcm2509324

**Published:** 2024-09-10

**Authors:** Attila Nemes, Árpád Kormányos, Dorottya Lilla Olajos, Alexandru Achim, Zoltán Ruzsa, Nóra Ambrus, Csaba Lengyel

**Affiliations:** ^1^Department of Medicine, Albert Szent-Györgyi Medical School, University of Szeged, H-6725 Szeged, Hungary

**Keywords:** left ventricular, twist, prognosis, speckle-tracking, three-dimensional, echocardiography, healthy

## Abstract

**Background:**

The left ventricular (LV) rotational mechanics are of particular importance in the function of the LV. The rotational movement is the consequence of the arrangement of the subepicardial and subendocardial muscle fibers. These muscle fibers are perpendicular to each other, their contraction creates a characteristic motion. The aim of the present study was to examine the prognostic impact of LV twist assessed by three-dimensional speckle-tracking echocardiography (3D-STE) in healthy circumstances.

**Methods:**

302 healthy adults participated in the study, 181 subjects were excluded due to certain reasons (LV could not be analysed during 3D-STE, subjects were unidentifiable, or lost to follow-up). 121 subjects were involved in the final analysis (mean age of 33.1 ± 12.3 years, 75 males), who were willing to be examined on a voluntary basis.

**Results:**

During a mean follow-up of 7.93 ± 4.21 years, 11 healthy adults suffered a cardiovascular event including 2 cardiac deaths. Using receiver operating characteristic analysis, LV twist ≥14.65 degrees as assessed by 3D-STE proved to be significantly predictive regarding the cardiovascular event-free survival (area under the curve 0.70, specificity 70%, sensitivity 65%, *p* = 0.028). Subjects with LV twist ≥14.65 degrees had higher basal and apical rotations and a significantly higher ratio of these individuals developed cardiovascular events compared to cases with LV twist <14.65 degrees. Subjects with cardiovascular events had lower LV global longitudinal strain, higher basal LV rotation and twist and the ratio of subjects with LV twist ≥14.65 degrees was elevated as compared to cases without events.

**Conclusions:**

3D-STE-derived LV twist independently predicts future cardiovascular events in healthy adults.

## 1. Introduction

Rotational mechanics of the left ventricle (LV) play an important role in the function of the LV [1, 2, 3, 4, 5]. The LV base rotates in a clockwise direction, while the apex of the LV moves in a counterclockwise direction during systole resulting in their net difference called LV twist. The physiology of this movement is based on the subepicardial and subendocardial muscle fibers running perpendicular to each other, their contraction creates a characteristic form of LV motion responsible for up to 40% of the ejection [1, 2, 3, 4, 5]. Although its physiological importance is known, several studies have been conducted recently analyzing its clinical significance even under healthy circumstances. In addition, further investigations are required to confirm its prognostic value. Three-dimensional (3D) speckle-tracking echocardiography (STE) seems to be an optimal method to determine its significance in real clinical settings due to its non-invasive and easy-to-perform nature [6, 7, 8, 9]. Therefore, the aim of the present study was to examine the prognostic significance of 3D-STE-derived LV twist in healthy adults.

## 2. Methods

### 2.1 Subject Population 

**Fig. 1.**
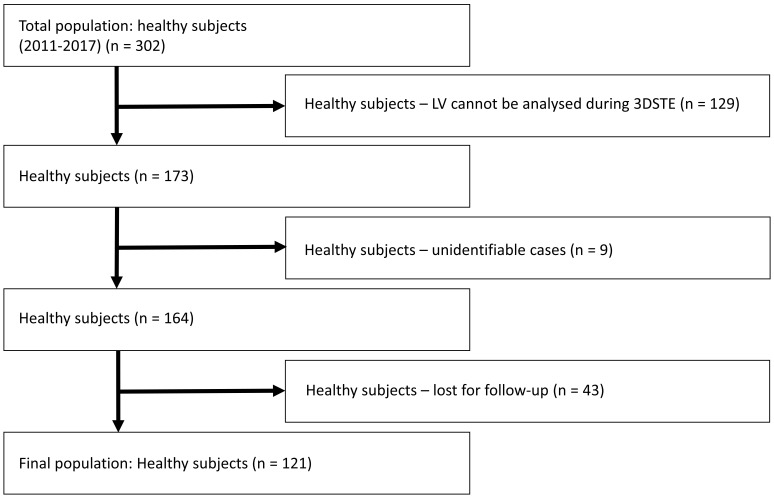
**Flowchart of the study with the total population and exclusions due to certain reasons**. Abbreviation: LV, left ventricle; 3D-STE, three-dimensional speckle-tracking echocardiography.

302 healthy adults participated in the present study, 181 subjects were excluded due to certain reasons (LV could not be analysed during 3D-STE, subjects were unidentifiable, or lost to follow-up). The final analysis involved 121 healthy volunteers (mean age of 33.1 ± 12.3 years, 75 males) (Fig. 1). Subjects were enrolled in the study between 2011 and 2017, and standard 12-lead electrocardiography (ECG), physical and laboratory tests, and two-dimensional (2D) Doppler echocardiography extended with 3D-STE were obtained. No abnormality was found with these tests, all findings were within the normal ranges. Exclusion criteria included any known disorder or pathology, obesity or any positive result with our tests. None of the subjects were athletes or participated in extensive training 2 weeks prior to enrolment. No medication or drug was taken by any individuals. The present retrospective study is part of the ‘Motion Analysis of the heart and Great vessels bY three-dimensionAl speckle-tRacking echocardiography in Healthy subjects’ (MAGYAR-Healthy) Study, in which, among other aims, diagnostic and prognostic values of 3D-STE-derived parameters were examined in real clinical settings in healthy individuals (‘Magyar’ means ‘Hungarian’ in the Hungarian language). The Institutional and Regional Human Biomedical Research Committee of University of Szeged, Hungary approved the study under the registration number of 71/2011 (prolonged on February 20, 2023). The study was conducted in accordance with the Declaration of Helsinki (revised in 2013) and all participants gave informed consent.

### 2.2 Follow-up Data

The primary outcome of the study was cardiovascular mortality, including sudden cardiac death and hospitalization to perform an invasive procedure, due to acute heart failure, angina pectoris, thrombosis, embolisation or arrhythmia. Primary outcome data were based on hospital recordings or autopsy reports.

### 2.3 Two-Dimensional Doppler Echocardiography

Toshiba Artida™ (Toshiba Medical Systems, Tokyo, Japan) echocardiography device was used in all cases. During the tests, all healthy subjects were lying on their left side. At this time, a wideband PST-30BT (1–5 MHz) phased-array transducer attached to the device was used for grayscale harmonic images and loops from the usual views. Usual parasternal and apical four- (AP4CH) and two-chamber (AP2CH) views were used for the determination of left atrial and LV sizes, volumes and ejection fraction (EF) [10]. Stenoses and regurgitations were excluded in the case of all valves with visual examination with the help of colour Doppler echocardiography.

### 2.4 Three-Dimensional Speckle-Tracking Echocardiography

**Fig. 2.**
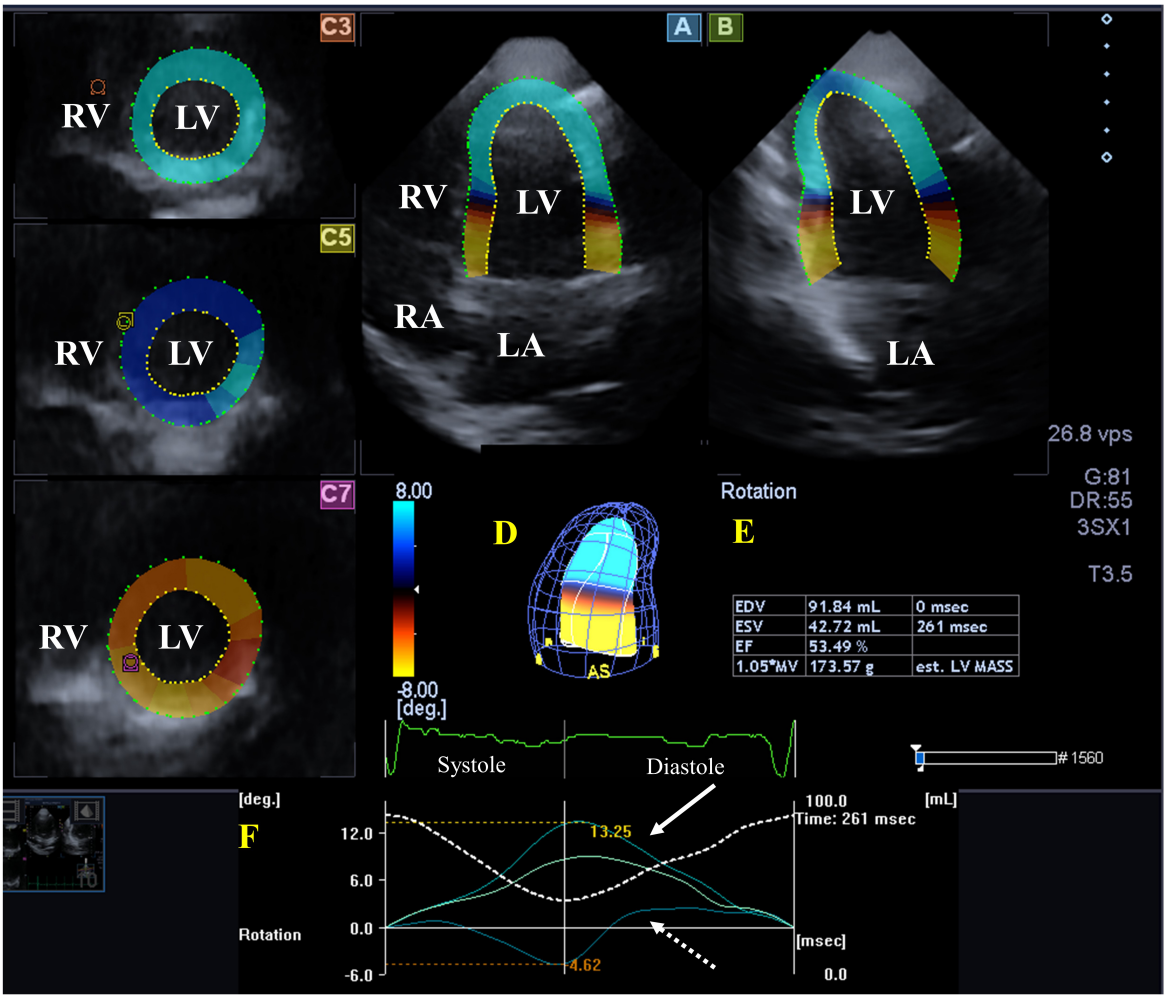
**Analysis of the left ventricle (LV) from a three-dimensional (3D) speckle-tracking echocardiographic dataset**. (A) Apical four-chamber long-axis view, (B) apical two-chamber long-axis view and (C3) apical, (C5) mid-ventricular, and (C7) basal LV short-axis views. A virtual 3D cast of the LV (D), LV volumetric data respecting the cardiac cycle (E), LV apical and basal rotations (coloured lines) and time-LV volume changes (dashed white line) during the cardiac cycle (red F) are presented in a healthy individual. Abbreviations: EDV, end-diastolic volume; ESV, end-systolic volume; EF, ejection fraction; MV, myocardial volume; msec, millisecond; vps, voxel per second; MASS, mass; LV, left ventricle; LA, left atrium; RV, right ventricle; RA, right atrium.

The tests continued with 3D-STE after transducer replacement to a PST-25SX (1–4 MHz) matrix phased-array transducer. Then 3D datasets were acquired following image optimization by adjusting gain, magnitude, etc. In all cases, for optimal images, 6 subvolumes, which shape resembled a wedge, focused on the LV were collected within 6 consecutive cardiac cycles, when the subject held her/his breath and ECG showed a constant RR interval. Data analysis was performed at a later date offline by Wall Motion Tracking software version 2.7 (Toshiba Medical Systems, Tokyo, Japan). All datasets were depicted in 3 short-axis views representing apical, midventricular and basal LV regions and in AP4CH and AP2CH long-axis views. For the creation of a 3D LV cast, the LV endocardium was manually defined in all cases at the mitral valve/LV base edges and at the LV apex on AP4CH and AP2CH views, and then a sequential analysis was performed forming a 3D virtual cast of the LV. Then, from several functional parameters, LV basal and apical rotations and LV twist were selected from the options offered by the software (Fig. 2) [6, 7, 8, 9].

### 2.5 Statistical Analysis

For continuous and dichotomous variables mean ± standard deviation and frequency/percentage (%) forms were used, respectively. A Kolmogorov–Smirnov test was used to check whether the data were normally distributed, in cases of a normal distribution, Student’s *t*-test was used and Mann–Whitney–Wilcoxon test was used in cases of non-normal distribution. Fisher’s exact test was used for the analysis of dichotomous variables. The predictive power of LV twist was established by a receiver operating characteristic (ROC) analysis and the area under the curve with sensitivity and specificity data were obtained. Kaplan–Meier life table estimates of survival were performed to summarize the follow-up. Long-rank test was obtained to test differences in survival rates between the groups. Statistical significance was present in case of *p* < 0.05 and all tests proved to be two-sided. Intraobserver and interobserver reproducibility was assessed by the calculation the intraclass correlation coefficient (ICC). For statistical analysis, SPSS software (version 22, SPSS Inc., Chicago, IL, USA) was used.

## 3. Results

### 3.1 Clinical and Demographic Data

Demographic and clinical data are shown in Table 1. Subjects with cardiovascular events and cases with LV twist ≥14.65 degrees were older.

**Table 1.** **Clinical, echocardiographic and follow-up data of healthy adults**.

	All subjects	LV twist <14.65°	LV twist ≥14.65°	No event	Event
No. of patients	121	83 (69)	38 (31)	110 (91)	11 (9)
Males (%)	62 (51)	42 (51)	20 (53)	56 (51)	6 (55)
Age (years)	30.7 ± 11.7	29.2 ± 10.2	34.1 ± 14.1*	29.1 ± 9.6	47.4 ± 17.5†
Two-dimensional echocardiography					
LV-EDD (mm)	48.2 ± 3.6	48.1 ± 3.7	48.5 ± 3.3	48.1 ± 3.6	48.7 ± 3.5
LV-EDV (mL)	106.5 ± 21.6	105.9 ± 22.8	107.9 ± 18.9	106.2 ± 22.0	109.5 ± 18.3
LV-ESD (mm)	31.8 ± 3.2	31.6 ± 3.5	32.2 ± 2.5	31.8 ± 3.4	31.9 ± 1.4
LV-ESV (mL)	35.8 ± 8.8	35.2 ± 9.4	37.2 ± 7.2	35.7 ± 9.0	37.5 ± 6.5
IVS (mm)	9.0 ± 1.6	9.0 ± 1.7	8.9 ± 1.5	8.9 ± 1.6	9.7 ± 2.0
LV-PW (mm)	9.0 ± 1.7	9.0 ± 1.8	9.0 ± 1.4	9.0 ± 1.7	9.6 ± 1.9
LV-EF (%)	66.5 ± 5.3	66.8 ± 5.9	65.8 ± 3.6	66.5 ± 5.4	65.9 ± 4.9
Three-dimensional speckle-tracking echocardiography					
LV-EDV (mL)	86.9 ± 22.4	87.9 ± 23.0	84.7 ± 21.1	86.9 ± 22.3	86.4 ± 24.5
LV-ESV (mL)	36.5 ± 10.0	37.1 ± 9.3	35.1 ± 11.5	36.4 ± 9.8	37.6 ± 13.2
LV-EF (%)	58.0 ± 5.1	57.6 ± 4.3	58.8 ± 6.5	58.1 ± 5.1	56.9 ± 5.6
LV-mass (g)	159.8 ± 30.4	159.6 ± 29.3	160.2 ± 33.1	158.6 ± 29.2	171.8 ± 40.6
LV-GLS (%)	–16.0 ± 2.3	–16.0 ± 2.1	–16.2 ± 2.7	–16.2 ± 2.3	–14.6 ± 1.8†
Basal LV rotation (°)	–4.2 ± 2.1	–3.6 ± 1.7	–5.5 ± 2.5*	–4.1 ± 1.9	–5.8 ± 3.3†
Apical LV rotation (°)	8.9 ± 3.6	7.4 ± 2.8	12.1 ± 3.2*	8.8 ± 3.7	9.9 ± 2.4
LV twist (°)	13.1 ± 4.1	11.0 ± 2.8	17.6 ± 2.3*	12.9 ± 4.0	15.7 ± 4.4†
LV twist time (ms)	351.8 ± 136.2	351.9 ± 139.9	351.4 ± 129.7	353.6 ± 138.9	333.7 ± 110.7
Pts with LV twist ≥14.65°	83 (69)	0 (0)	38 (100)*	31 (28)	7 (64)*
Events					
Subjects with events (%)	11 (9)	4 (5)	7 (18)*	0 (0)	11 (100)†
Subjects with death (%)	2 (2)	1 (1)	1 (3)	0 (0)	2 (18)†

†*p* < 0.05 vs. no events; *p < 0.05 vs. LV twist <14.65°. Abbreviations: LV, left ventricular; EDD, end-diastolic diameter; EDV, end-diastolic volume; ESD, end-systolic diameter; ESV, end-systolic volume; IVS, interventricular septum; PW, posterior wall; EF, ejection fraction; GLS, global longitudinal strain.

### 3.2 Two-Dimensional Echocardiography

None of the routine 2D echocardiography-derived parameters differed between the groups as presented in Table 1. Subjects with cardiovascular events had (non-significantly) thicker LV posterior wall and interventricular septum.

### 3.3 Left Ventricular Twist

**Fig. 3.**
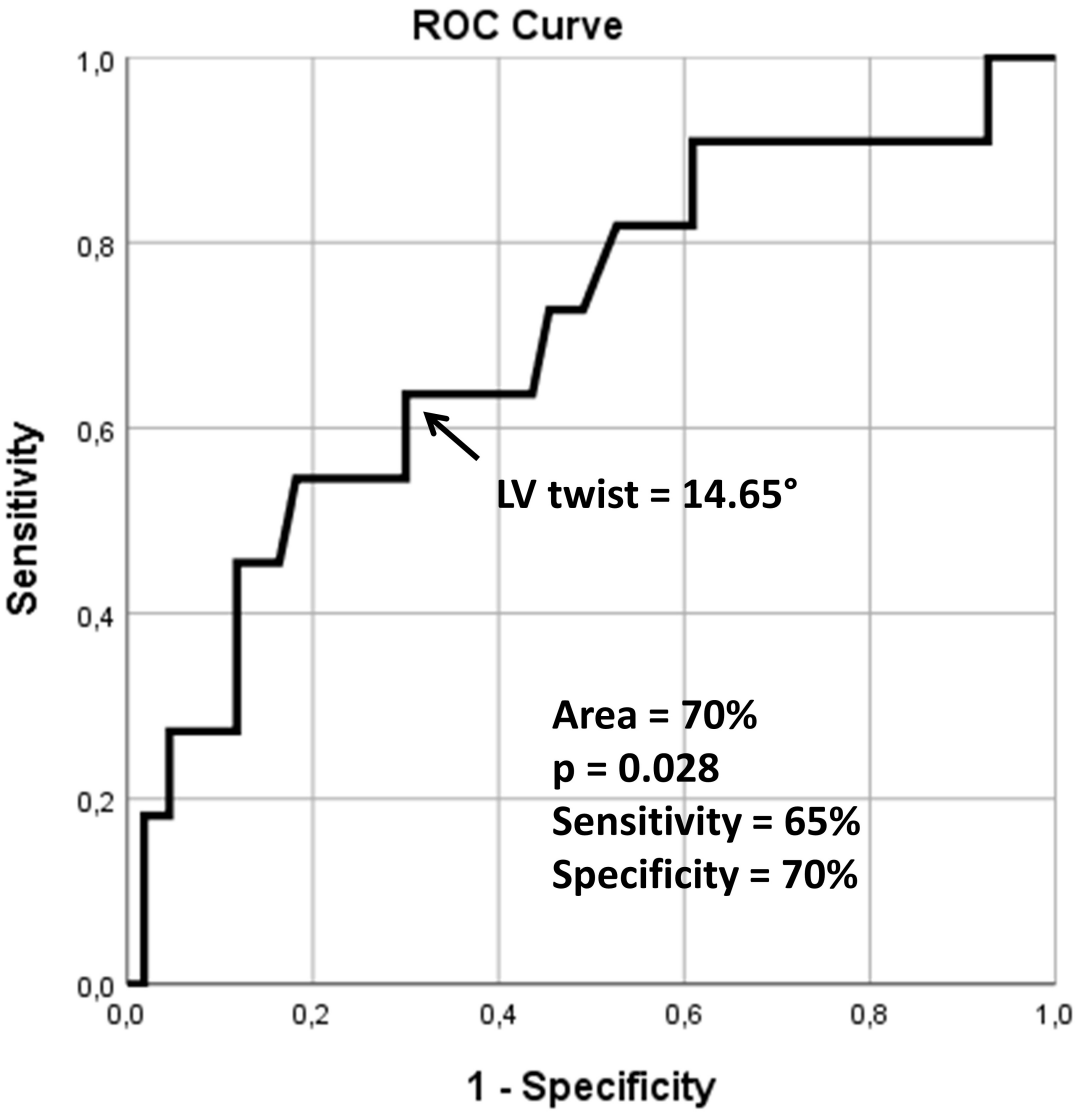
**Receiver operating characteristic analysis illustrating the diagnostic accuracy of left ventricular twist as assessed by three-dimensional speckle-tracking echocardiography in predicting cardiovascular morbidity and mortality**. ROC, receiver operating characteristic; LV, left ventricular.

**Fig. 4.**
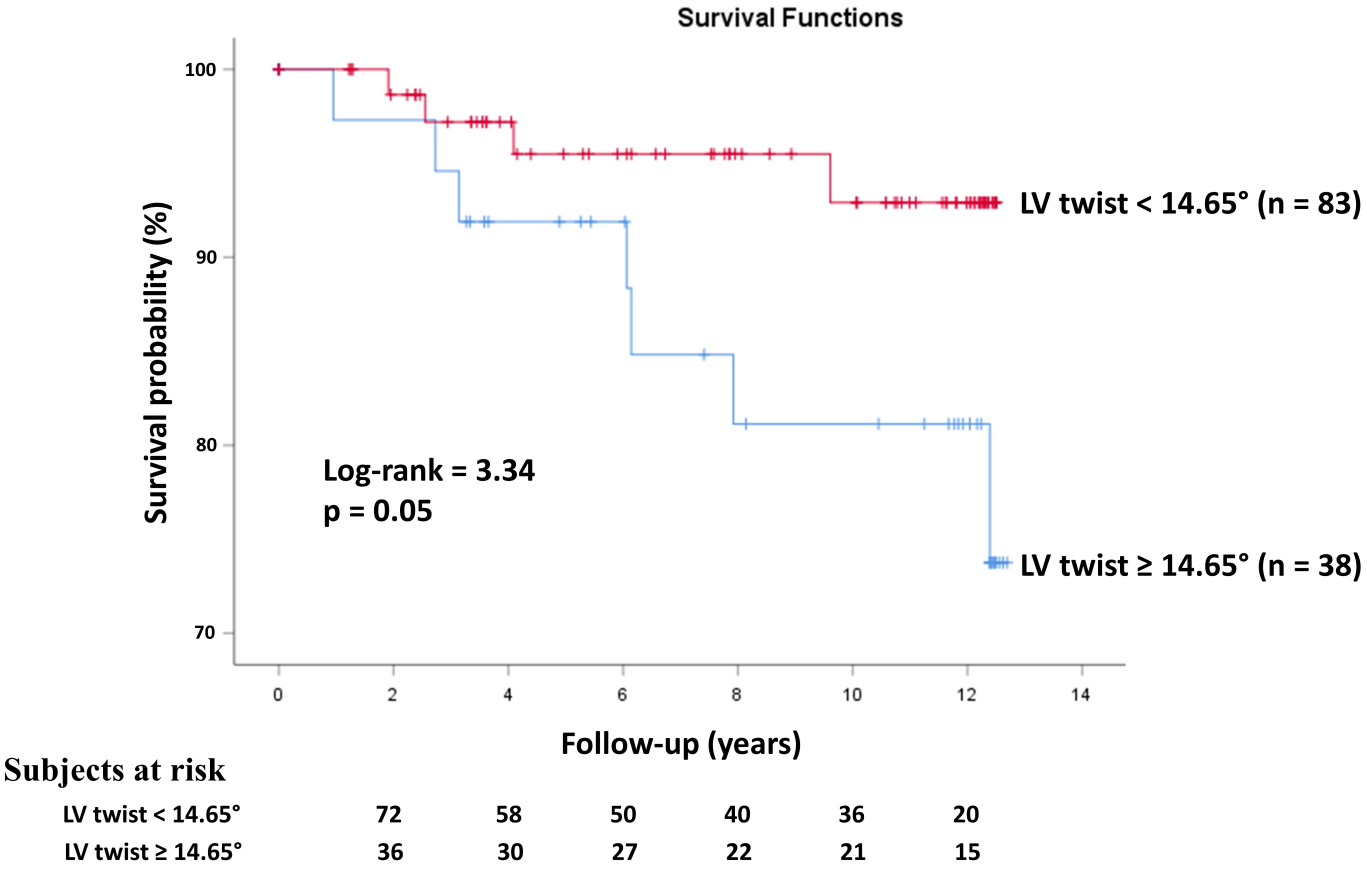
**Kaplan–Meier survival curves illustrating the predictive role of three-dimensional speckle-tracking echocardiography-derived left ventricular twist**. LV, left ventricular.

Using ROC analysis, 3D-STE-derived LV twist ≥14.65 degrees was a significant predictor of cardiovascular event-free survival (sensitivity 65%, specificity 70%, area under the curve 0.70, *p* = 0.028) (Fig. 3). The Kaplan–Meier cumulative survival curve illustrating the predictive role of 3D-STE-derived LV twist is presented in Fig. 4.

### 3.4 Three-Dimensional Speckle-Tracking Echocardiography

The mean frame rate was 31 ± 2 fps. Subjects with LV twist ≥14.65 degree had higher basal and apical LV rotations and a significantly higher ratio of these individuals developed cardiovascular events compared to subjects with LV twist <14.65 degrees. Subjects with cardiovascular events had lower LV global longitudinal strain (GLS), higher basal LV rotation and twist and the ratio of subjects with LV twist ≥14.65 degrees was higher in cases of subjects with cardiovascular events as compared to subjects with no events. All other 3D-STE-derived variables were similar between the groups as seen in Table 1.

### 3.5 Events

During a mean follow-up of 7.93 ± 4.21 years, 11 healthy adults suffered a cardiovascular event including 2 cardiac deaths. In 3 out of 11 cases acute heart failure and angina pectoris developed, 3 other subjects had undergone invasive procedures (percutaneous coronary intervention with stent-implantation), deep vein thrombosis/pulmonary embolism was found in 2 patients and 1 patient was hospitalized due to paroxysmal supraventricular tachycardia.

### 3.6 Intraobserver and Interobserver Reproducibility

Intraobserver and interobserver ICCs of LV twist proved to be 0.84 and 0.83, respectively.

## 4. Discussion

LV contractility represented by LV strains and LV rotational mechanics play a fundamental role in LV function [11, 12, 13, 14, 15]. In systole, the LV shortens in a longitudinal direction represented by LV longitudinal strain (LS), narrows in a circumferential direction represented by LV circumferential strain and thickens in a radial direction represented by LV radial strain [16]. In addition, the rotational mechanics of the LV play a significant role in optimizing its pumping function as well. This is due to the special LV myocardial architecture: there are two perpendicular left- and right-handed muscle bands in the subepicardium and subendocardium of the LV. The torque of the subepicardial one is larger, so its effect prevails. Accordingly, although the shortening of the LV muscle fibers during the heart cycle is about 15–20%, the LV ejection fraction (EF) is normally 60–65% [3].

Clinically, the prognostic impact of echocardiography-derived LV-EF and LV-GLS is well-documented, but the predictive value of LV rotational mechanics needs further investigation [17, 18, 19]. For instance, increased LV twist was predictive of non-sustained ventricular tachycardia in hypertrophic cardiomyopathy patients [17]. In another study, LV twist was found to be a predictor of preserved LV function after surgery in severe mitral regurgitation [18]. The presence of LV torsion independently predicted peak oxygen uptake during exercise and its impairment proved to be predictive of a reduced functional capacity [19]. LV twist was found to be a predictor of mortality in aortic valve stenosis as well [20]. Although in several scientific works, 2D-STE was used for the detection and calculation of LV rotational parameters [21], according to recent recommendations, 2D-STE is not recommended for measuring LV apical and basal rotations [22]. 3D-STE combines the advantage of STE and 3D echocardiography allowing visualization of the LV as a heart chamber with 3D features for simultaneous assessment of volumes, strains and rotational parameters using the same 3D virtual LV cast respecting the cardiac cycle [6, 7, 8, 9]. 3D-STE is an easy-to-learn and easy-to-implement technique, which is validated for the determination of LV twist [23, 24, 25], and normal reference values are also available [26].

The prognostic value of 3D-STE-derived LV twist on survival is a less examined phenomenon [20]. According to the presented findings, it could be stated that increased LV twist, mostly due to elevated LV basal rotation, shows associations with increased risk of cardiovascular events in a healthy population during a 12-year follow-up period. In subjects with cardiovascular events, not only was the LV-GLS, a known prognostic factor, reduced, but LV twist proved to be increased due to elevated LV basal rotation. Moreover, two-thirds of the events were present in subjects with increased LV twist. It should be emphasized that these associations were present in an apparently healthy population. One might rightly ask what could explain this. As we know, it is the subepicardial layer in the LV that determines the direction of LV rotation. In cases of abnormalities like ischaemia affecting the subendocardium, LV overrotation is expected to be detected [3]. Accordingly, this overrotation, which can be determined by 3D-STE, has a strong prognostic value. Moreover, in correspondence with the previous findings, LV-GLS and age showed associations with LV rotational mechanics as well [27, 28]. These results should be assessed in the light of the fact that no significant abnormalities could be demonstrated with routine examinations in the healthy individuals. However, it cannot be excluded with certainty that there was no latent pathology that required hospitalization and/or care later during the long-term follow-up, as confirmed by the results (invasive care, death). Further investigations are warranted in a larger healthy population to confirm the presented findings.

## 5. Limitations

The most important limitations are listed below:

- The image quality is still a significant limitation of 3D-STE, which may have had significant effects on the results obtained.

- Only the prognostic impact of LV twist was analyzed, the predictive role of other 3D-STE-derived parameters on survival was not examined.

- The concept of healthiness was pronounced based on the results of routine examinations in individuals who claimed to be complaint-free. During follow-ups, significant cardiovascular events occurred in 9% of the cases, which can mean that some of the subjects could have had latent diseases.

- When evaluating the obtained results, it should be taken into account that the study design was retrospective. In addition, clinical and echocardiographic data of a relatively small number of healthy individuals were analysed using a relatively new imaging technology.

## 6. Conclusions

3D-STE is suitable for non-invasive evaluation of LV rotational mechanics. In cases of cardiovascular events, LV twist was increased as a consequence of increased LV basal rotation, which can be explained by abnormalities such as subendocardial ischaemia leading to LV overrotation. Based on the results of a prolonged follow-up, it can be said that 3D-STE-derived LV twist independently predicts future cardiovascular events in healthy adults.

## Availability of Data and Materials

This author takes responsibility for all aspects of the reliability and freedom from bias of the data presented and their discussed interpretation.
